# Evaluating Pain and Analgesia Effectiveness Following Routine Castration in Rabbits Using Behavior and Facial Expressions

**DOI:** 10.3389/fvets.2022.782486

**Published:** 2022-02-28

**Authors:** Amy L. Miller, Jasmine M. Clarkson, Caroline Quigley, Vikki Neville, Caroline Krall, Annika Geijer-Simpson, Paul A. Flecknell, Matthew C. Leach

**Affiliations:** ^1^School of Natural and Environmental Sciences, Newcastle University, Newcastle upon Tyne, United Kingdom; ^2^Institute of Biodiversity, Animal Health and Comparative Medicine, College of Medical, Veterinary & Life Sciences, University of Glasgow, Glasgow, United Kingdom; ^3^Bioresearch and Veterinary Services, University of Edinburgh, Edinburgh, United Kingdom; ^4^Bristol Veterinary School, University of Bristol, Bristol, United Kingdom; ^5^Department of Molecular & Comparative Pathobiology, Johns Hopkins University School of Medicine, Baltimore, MD, United States; ^6^Comparative Biology Centre, Newcastle University, Newcastle upon Tyne, United Kingdom

**Keywords:** rabbit, pain, behavior, analgesia, rabbit grimace scale

## Abstract

Prevention of pain in rabbits is a priority for both welfare and validity of scientific data. We aimed to determine if the rabbit grimace scale (RbtGS) could be used as a viable, rapid assessment tool in two breeds of rabbit, Dutch belted (DB) and New Zealand white (NZW), following orchidectomy, as an adjunct to behavioral analysis. All animals received analgesia. Rabbits were filmed and their behavior was recorded at multiple time points pre- and post-orchidectomy. Observers then scored specific pain associated behaviors for analysis. Time matched footage was also scored using the rabbit grimace scale (RbtGS). Following surgery, rabbits showed significant increases in the duration spent displaying key pain associated behaviors at 1 and 5 h post-surgery. DB rabbits that received low dose meloxicam (0.2 mg/kg) showed significantly more pain behaviors at 1 and 5 h post-surgery compared to those administered a combination of higher dose meloxicam (0.6 mg/kg) and a lidocaine/bupivacaine local infusion. DB rabbits showed an increase in RbtGS score at both 1 and 5 h post-surgery. In the NZW rabbits, an increase in RbtGS score was only observed at 1 h post-surgery. Using behavioral analysis as the gold standard for comparison, the RbtGS was an effective means of determining when rabbits are painful following orchidectomy. Higher dose meloxicam (0.6 mg/kg) combined with local anesthetic was a more effective method of reducing pain, compared to lower dose meloxicam (0.2 mg/kg) alone.

## Introduction

Rabbits are a popular pet and a common laboratory and farm species globally. In the UK, over 900,000 rabbits are kept as pets (1) and over 11,000 used in regulated scientific procedures per annum (2). Globally, rabbits are the 4th most farmed livestock species and provide over 1.5 million tons of meat annually (3, 4). The majority of these animals will undergo at least one potentially painful procedure during their lifetime, with routine neutering of companion animals being one of the most common. Unalleviated pain not only compromises welfare, but is considered inappropriate by the public, compromises the quality and reliability of data collected from laboratory animals (5), and decreases production parameters in farmed animals. Despite the large numbers of rabbits undergoing such procedures, there is limited research on developing validated means of assessing pain in this species (6), thus little is known about the actual effectiveness of the analgesic drugs available for use in rabbits. This lack of previous research, on validated means of pain assessment, has three interlinked consequences. Firstly, analgesia administration appears to remain low (6, 7) and is lower compared to other large laboratory species, e.g., pigs, sheep, and non-human primates (8). Secondly, we cannot be confident about the effectiveness of the analgesics administered following potentially painful procedures (6). Finally, it is not possible to develop new and more effective means of relieving pain with certainty. For example, there has been an increasing move in recent years toward multimodal analgesia (i.e., provision of lower doses of >1 analgesic from different drug classes) based on the assumption that this will provide more effective pain relief in rabbits, as it does in other species. In both man and other species, this approach has been shown to provide more effective analgesia by targeting more than one pain pathway (9–12). The approach can also enable use of lower doses of the analgesic agents when they are used in combination, reducing their associated side effects (13). Multimodal analgesia may have potential benefits in rabbits, since a single high dose of meloxicam alone (1 mg/kg followed by 0.5 mg/kg for 2 days post-surgery) provided relatively limited analgesia post-ovariohysterectomy in New Zealand white rabbits (14). In contrast, Goldschlager et al. (11) showed that buprenorphine (0.01 mg/kg) and meloxicam (0.1 mg/kg) in combination prevented a rise in fecal corticosterone metabolites (FCM) in New Zealand White rabbits. Additionally, these rabbits gained more weight in the 28 days following surgery than those that received only a single analgesic. Evaluating approaches such as this requires effective pain scoring systems.

Leach et al. (14) carried out one of the first studies to develop a pain assessment system for rabbits following ovariohysterectomy. This study demonstrated that a range of spontaneous behavioral changes occurred in the immediate hours following surgery, e.g., increased periods of inactivity coupled with the presence of abnormal behaviors such as twitching, wincing and staggering. However, behavioral pain assessment is highly labor intensive and time consuming, posing a significant limitation. An additional significant problem associated with behavioral assessment in rabbits is the freezing response commonly displayed in the presence of an observer. This can be overcome by remote viewing of animals, but this may not be practicable when monitoring for a protracted period, i.e., covering the full recovery from a surgical procedure. Therefore, an alternative method of assessing pain in rabbits is required.

Various grimace scales exist for evaluating pain in a variety of species (15–19), including rabbits (RbtGS) (20). Keating et al. (20) demonstrated that immediately following punch tattooing of the ears, RbtGS scores in New Zealand white rabbits increased, alongside changes in blood pressure and vocalizations. The application of the local anesthetic cream, EMLA, to the ears of the rabbits in advance of tattooing mitigated these changes. The Rabbit Grimace Scale (RbtGS) consists of 5 Facial Action Units (FAUs); orbital tightening, cheek flattening, pointed nose, whisker change and ear shape and position. A three-point scale is used to score the intensity which each of these FAUs are exhibited, with higher scores being associated with pain (20).

One of the benefits of grimace scale scoring is that it is significantly less time consuming than standard behavioral analysis, therefore has the potential to allow more rapid means of assessing pain and therefore screening of analgesics for their effectiveness. Since humans have a natural tendency to look at the faces of animals, rather than their body (21), assessing facial expression may also be an easier method to implement.

The primary aim of the current study was to determine if the Rabbit Grimace Scale (RbtGS) and Spontaneous Pain Behavior Scale (SPBS) provided an effective and rapid means of assessing pain following orchidectomy in rabbits. We also aimed to evaluate two methods of scoring the RbtGS; still images compared to scoring from video recordings to compare their effectiveness for scoring pain. Finally, we included two different multimodal regimes in two rabbit breeds. In Batch 1, use of meloxicam at the currently recommended dose rate (0.6 mg/kg) together with local infiltration of the surgical site with lidocaine and bupivacaine was evaluated in Dutch belted rabbits in comparison to meloxicam at a previously used dose (0.2 mg/kg) (22) as the sole analgesic agent. In Batch 2, the combination of meloxicam (0.6 mg/kg) and buprenorphine together with local infiltration of the surgical site with lidocaine and bupivacaine was evaluated in New Zealand White rabbits. As previous work has included a negative control group, i.e., a group which received no analgesia (14), we opted to avoid including animals with unalleviated pain and compared a previously recommended used analgesic regime (meloxicam, 0.2 mg/kg alone). We considered this would enable assessment of a range of degrees of post-operative analgesia in the different treatment groups.

## Materials and Methods

All procedures were conducted in accordance with the Animals (Scientific Procedures) Act 1986 (PPL 60/4431), European Directive 2010/63 and with the approval of the Newcastle University Animal Welfare Ethical Review Body. This manuscript was prepared in accordance with the ARRIVE guidelines. This study employed a “rescue analgesia” policy whereby if any animal displayed >4 pain behaviors within 5 min (assessed by a veterinarian who was not otherwise involved in the study), 0.05 mg buprenorphine s.c. was administered immediately. No animals required this intervention. All animals recovered uneventfully from surgery.

### Animals and Husbandry

This study used two separate batches of rabbits (Batch 1 and Batch 2). Batch 1 contained 16 male Dutch belted rabbits (Harlan, USA) aged 6 months at the start of the study. Batch 2 contained 16 male New Zealand white rabbits (Charles River, UK) aged 5 months at the beginning of the study. The animals were free from any common pathogens in accordance with FELASA health monitoring recommendations. These two breeds were selected for study as they are a typical pet breed (Dutch-Belted) and typical laboratory breed (New Zealand White). On arrival, rabbits were individually housed in floor pens (1 × 2 m) with sawdust bedding (Datesand, UK). Each pen contained a cardboard box acting as both a shelter and a platform, a cardboard tube (large enough for the rabbit to enter), a cat litter tray and chew blocks for enrichment (Datesand, UK). Although kept in individual pens, rabbits were able to see, hear, smell and touch conspecifics through adjoining mesh pen walls. Food (Raba complete rabbit food, SDS Ltd) and tap water were provided *ad-libitum*. Additionally, hay and cabbage or carrots were provided daily. The room was maintained at 21 ± 2°C, humidity 50% and a 12/12 h light/dark cycle (lights on at 07:00). A 14-day acclimation period was given before the start of the study for each Batch. During this time, the rabbits were habituated to the general daily activity of the animal care staff, handling, weighing, the presence of the observers, and the video monitoring equipment.

The rabbits were housed individually to prevent fighting between intact males and to prevent any changes in behavior or facial expressions because of transient separation from their pen mates during observations (see below). The rabbits were then housed in groups of 2–4 (with neighboring rabbits), at 2–3 weeks post castration, in preparation for rehoming as domestic pets. Groups of rabbits were housed in larger pens (2 × 2 m for pairs or 3 × 2 m for >3 rabbits) in the same manner as described above. Before rehoming, all rabbits underwent a veterinary examination, were formally released from the controls of the Animals (Scientific Procedures) Act 1986 and vaccinated against myxomatosis and viral haemorrhagic disease.

### Baseline Recordings

**Figure 1:**
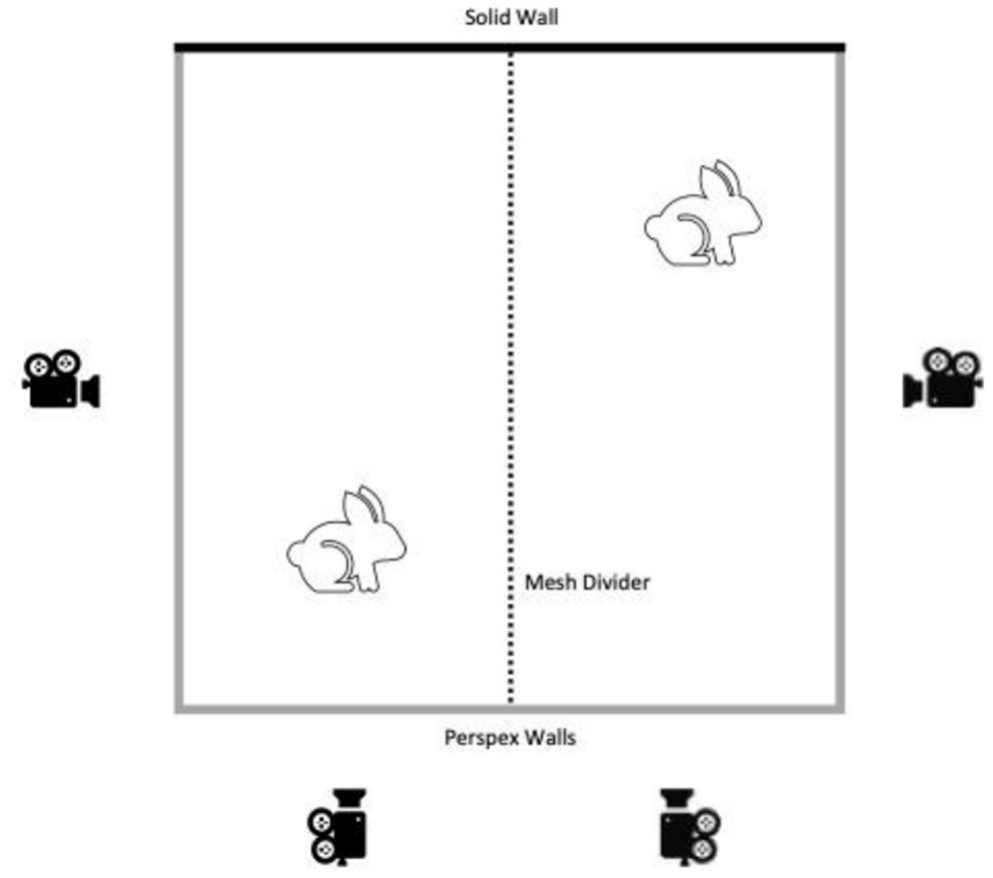
Set up of the filming area, showing pen layout and camera position.

The week before surgery, all rabbits were placed individually in the filming arena (2 × 2 m: NKP cages, UK) which contained only sawdust bedding. The arena consisted of 3 clear Perspex sides with a mesh divider splitting into two filming pens (1 × 2 m). Two HD video cameras (DCR-VX2100E, Sony, Japan) were placed at a fixed distance from each of the two Perspex walls of each filming pen and remotely operated from outside the room at all times (Figure 1). The rabbits were allowed 5 min to habituate to placement in the filming pen and then filming commenced for 15 min. A companion rabbit, which was not scheduled for surgery at that time, was placed in the adjoining pen, separated by the mesh wall, to simulate a home pen environment. Each animal was recorded twice at baseline; once in the morning (Baseline AM) and once in the afternoon (Baseline PM). These two recording times were selected to be time matched with the post-surgery recording times. On the same day, animals in Batch 2 also had home pen footage recorded during the dark phase of their photoperiod (19:00–07:00) by placing GoPro Hero 5.0 cameras above each home pen to record the activity of each animal.

### Treatment Allocation

The two treatments of meloxicam only or multimodal analgesia (see Table 1) were randomly allocated to the rabbits (*n* = 8 per treatment group) in each Batch using a random number generator (www.random.org).

**Table 1:** Treatment group allocations for Batch 1 (Dutch Belted rabbits) and Batch 2 (New Zealand White rabbits).

**Batch**	**Treatment** **group 1**	**Treatment group 2**
1	Meloxicam 0.2mg/kg s.c.	Meloxicam 0.6 mg/kg s.c. + Lidocaine (0.4 mg/kg)/Bupivacaine (0.2 mg/kg) local infiltration
2	Meloxicam 0.2 mg/kg s.c	Meloxicam 0.6 mg/kg s.c. + buprenorphine 0.03 mg/kg + Lidocaine (0.4 mg/kg)/Bupivacaine (0.2 mg/kg) local infiltration

### Anesthesia and Analgesia Controls

Anesthesia and analgesia (AA) only control data were collected from rabbits in Batch 2. Video recordings were time of day matched with Baseline AM and Baseline PM recordings. Therefore, these rabbits acted as within-subjects' controls for the effect of treatment only. On the day of AA control data collection, rabbits received their allocated dose of meloxicam or multimodal analgesia (Table 1). Thirty minutes later, anesthesia was induced with i.v. propofol (10 mg/kg). Rabbits were then placed on a heated blanket (Harvard apparatus, Edenbridge, UK), intubated, and anesthesia maintained with sevoflurane (4–6%) in oxygen (4 L/min). Rabbits were placed in dorsal recumbency, the scrotal area shaved, and anesthesia maintained for 30 min. Rabbits then recovered in an incubator (25°C) for 30 min before being placed into the filming pen. AA-AM and AA-PM video recordings were then carried out, time matched with Baseline AM and Baseline PM, in the same manner as described above. That evening, the rabbits were also recorded in their home pens, as described above, to record dark phase activity of each animal. A minimum of a 7 day wash out period was then given prior to surgery.

### Surgery

On the morning of surgery, rabbits were transported to theater using a pet carrier. All rabbits received their allocated dose of meloxicam or multimodal analgesia (Table 1) 30 min before anesthesia. Surgery began between 09:00 and 10:30 and, anesthesia was induced and maintained as described above. Following shaving, the scrotum was sprayed with chlorhexidine (Hydrex Derma spray, Adam Healthcare, Leeds, UK) and the skin was infiltrated with saline (Group 1) or local anesthetic (Group 2) followed by orchidectomy. Surgery was carried out using full aseptic technique. A 2 cm incision was made, testes were blunt dissected, and the cord infiltrated with saline (Group 1) or local anesthetic (Group 2). The testes were then clamped proximally to the point of infiltration, transfixed and ligated with 3.0 Vicryl. Once removal of the testes was completed, the dead space was closed and the skin incision repaired using a subcuticular closure technique, using 3.0 Vicryl. The same experienced surgeon carried out all procedures in both batches. No rabbit was anesthetized for more than 30 min. Following surgery, rabbits recovered in an incubator (25°C) for 1 h, where they received close monitoring by animal care staff. The rabbits were then transferred to the filming pen for the first post-surgery video recording. Daily wound checks and health monitoring were carried out until the wound had fully healed.

### Post-surgery Recordings

Following surgery, the filming process was repeated, as described above at various time points, with time of day matched with Baseline AM, Baseline PM, AA-AM, and AA-PM. For Batch 1, filming was carried out at 1, 5, 24, and 48 h post-surgery. For Batch 2, filming was carried out at 1, 5, 24, and 29 h post-surgery. Following data collection for Batch 1 at 48 h, we felt that it was important to study an intermediate time point in the second Batch. By 48 h behavior had largely returned to baseline levels so we took the opportunity to study a time matched (with respect to baseline) point to determine how long the acute painful effects of surgery lasted. Dark phase activity was again monitored for those rabbits in Batch 2 on the day of surgery and surgery day +1.

### Bodyweight and Food/Water Consumption

Individual body weight data were collected on Baseline, AA, Surgery day and Surgery day+1 for all rabbits. For Batch 2 only, this data was also collected on surgery day +2, together with food and water consumption measured at each time point, by weighing the water bottles and any food that remained in the pen.

### Behavioral Data Collection

Fifteen minutes of manual behavioral analysis was carried out, for each rabbit, at each time point by treatment and time-point blinded observers (one for Batch 1 and two for Batch 2) using Cowlog 2.0 software (23), and an ethogram based upon that of Leach et al. (14) (Table 2). All observers were fully trained before scoring began by scoring short rabbit sequences and comparing their scores to those made by an experienced observer. All observers showed above threshold (90%) consistency with the experienced observer ML. For dark phase activity, each video sequence was analyzed to determine the rabbit's location within the home pen every 5 min over the 12 h recording period. For analysis, the floor of the pen was divided into six equal-sized, distinct zones and activity was calculated by counting the frequency of transitions between zones.

**Table 2:** Ethogram used to score 15 min of individual rabbit behavior pre and post castration [Ethogram based upon (14)].

**Behavior**	**Behavior description**
Twitching	Rapid contraction of the back muscles
Flinching	Large, rapid movement of the body
Wincing	Rapid backwards rocking motion, accompanied by eye closing, and swallowing action
Staggering	Partial loss of balance
Falling	Complete loss of balance while moving
Pressing	Abdomen is pushed toward the floor
Arching	Back pushes upwards to create an inverted “U” shape to the body
Writhing	Contraction of flank muscles
Shuffling	Forwards movement, very slow pace
Active	Time spent engaging in physical pursuits
Inactive	Time spent with little or no motion
Composite pain (frequency)	Sum of the number of incidents following behaviors: arching, falling, flinching, twitching, pressing, staggering, wincing, writhing, shuffling
Composite pain (duration)	Total time spent displaying the following behaviors: arching, pressing, falling, writhing, shuffling

### Rabbit Grimace Scale (RbtGS)

The RbtGS was scored using two methods: still image scoring and video scoring. Scoring of the still images was carried out on images extracted from the 15 min HD videos recorded in the filming pen. An image was extracted on every occasion the rabbit's face was clearly visible, except for when the rabbits were grooming, eating, or sleeping. There was a minimum period of 30 s between subsequent extracted images. The images were cropped to leave only the face visible, to prevent bias in scoring linked to body posture (19). Two images of each rabbit, at each time point were then randomly selected for RbtGS scoring using a random number generator (www.random.org). The chosen images were then randomly re-ordered and inserted into a custom-designed excel spreadsheet for scoring. Participants (10 for Batch 1 and 7 for Batch 2) who were blind to all aspects of the experimental design and purpose scored the images using the RbtGS. Each participant was provided with a RbtGS manual for reference when scoring in the images. Prior to scoring the images from this study, participants scored a small number of rabbit images, and these scores were compared to those from an experienced observer (ML). Participant scores were consistent with those of the experienced observer. The images were scored for all 5 of the FAUs comprising the RbtGS on a 3-point scale; 0 = not present, 1 = moderately present, 2 = obviously present. On occasions where the observer was unable to score a particular FAU, they were asked to mark this as “not visible,” following Keating et al. (20).

Video scoring was used to simulate remote live scoring in a clinical context. The same videos that were used for the production of the still images were scored by treatment and time-point blinded observers in a randomized order, assigned using a random number generator (www.random.org). In Batch 1, the video files were watched and paused every 20 s and scored using the RbtGS as described above made at the instance in time. To reduce the time taken for analysis, in Batch 2, four 30 s video sequences from across the 15 min video were extracted and scored. These video sequences were selected at random from each video file. Scoring was done based on the cumulative impression given by the 20- or 30-s clip observed.

For both RbtGS scoring methods, a composite GS score was calculated by summing the scores for each of the 5 FAUs for each image or video sequence [following (20)].

### Statistical Analysis

All statistical analyses were conducted in R (v 3.5.1, R Core Team, www.r-project.org/) via R studio (version1.1.456, RStudio, PBC, 2009-2020,). Generalized linear mixed models via the lme4 package (24) were carried out to compare behaviors or RbtGS scores between the various time points and analgesic groups. This method was also used to compare zone transitions during the dark phase for Batch 2 rabbits. Bodyweight and change in food and water consumption was analyzed using a linear mixed model from the lme4 package. We used a likelihood-ratio test (LRT) between models, which calculates the difference in model deviance (χ2 distributed) when a predictor variable is removed. For the rabbits in Batch 2, Tukey *post-hoc* tests were carried out where appropriate. The different methods of assessment were not compared statistically due to the differing timings of data collection. The two breeds of rabbits have not been directly compared as they were studies in two independent batches. Results were considered statistically significant when *P* < 0.05.

In the RbtGS data set, there were a significant number of missing data points due to observers being unable to determine FAUs, due to orientation of the face. Instead of listwise deletion, missing data were simulated using the widely accepted method of multiple imputation in R using the mice package. Missing values were imputed using 50 iterations, generating five imputed data sets using predictive mean matching (25–27).This is the method of choice for complex, incomplete data sets, where predictive mean matching ensures imputed values are plausible if the assumption of normality is violated (25, 28). Rather than replacing missing data with a single mean/median value, this method instead uses the distribution of the observed data to estimate multiple possible values (e.g., five values) for the data points. This accounts for the uncertainty around the true value, obtaining unbiased estimates whilst accounting for variability.

## Results

### Composite Pain Behaviors

#### Batch 1 (Dutch Belted)

**Figure 2:**
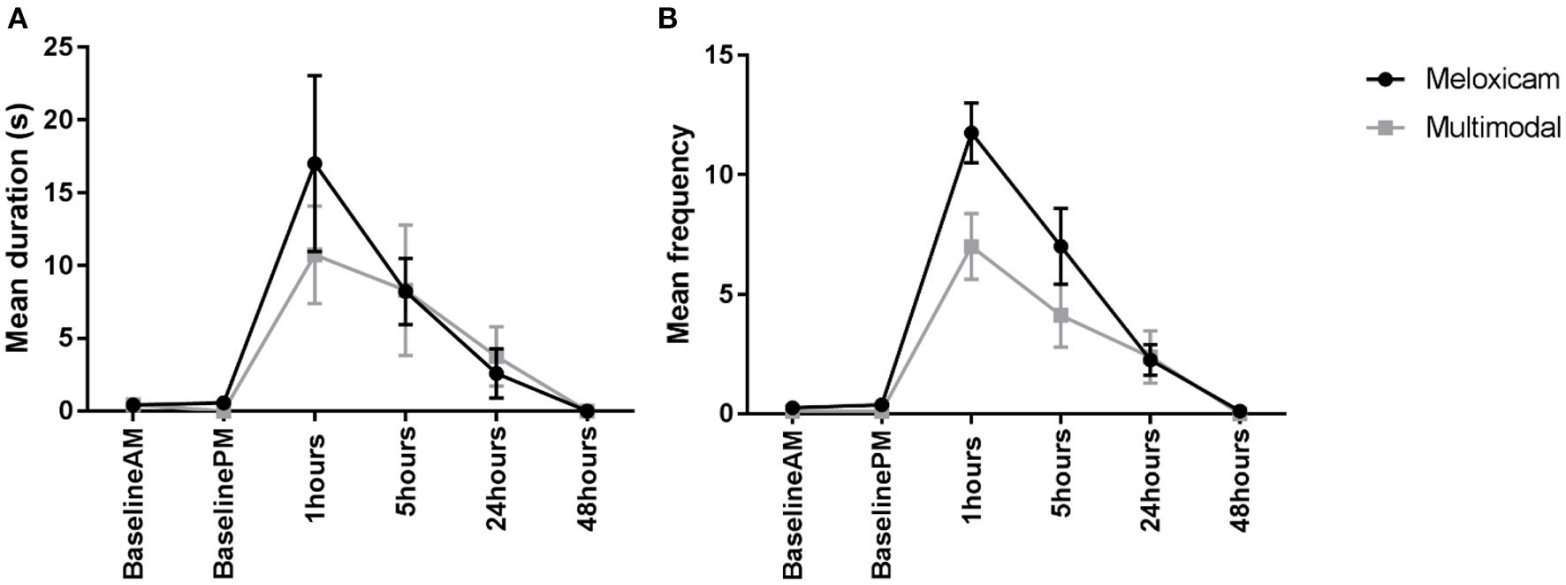
**(A)** The mean (±SEM) duration (s) of pain behaviors (arching, pressing, falling, writhing, and shuffling) over time, in Dutch Belted rabbits administered meloxicam alone or a multimodal analgesic regime (*n* = 8 per treatment group). **(B)** The mean (±SEM) frequency of pain behaviors (arching, falling, flinching, twitching, pressing, staggering, wincing, writhing, and shuffling) over time, in Dutch Belted rabbits administered meloxicam alone or a multimodal analgesic regime (*n* = 8 per treatment group).

For the duration of time spent displaying active pain behaviors (composite pain duration) during the morning observations (Baseline AM and 1, 24, and 48 h post-surgery), the only factor found to have a significant effect was time (X^2^ = 77.203, *p* < 0.001). Tukey pairwise comparisons revealed a greater duration spent displaying active pain behaviors 1 h post-surgery compared to all other morning observations, irrespective of the treatment group. Similarly, for the duration of active pain behaviors during the afternoon observations (Baseline PM and 5 h post-surgery), the only factor found to have a significant effect was time (X^2^ = 25.476, *p* < 0.001), with a greater duration spent displaying these behaviors at 5 h post-surgery. Again, this was irrespective of the treatment group, with no significant difference found between the meloxicam alone and multimodal treatment groups (Figure 2A).

For the frequency of active pain behaviors displayed (composite pain frequency) during the morning observations, there was a significant main effect of treatment (X^2^ = 5.5015, *p* = 0.025), with rabbits administered meloxicam alone exhibiting significantly more pain behaviors compared to rabbits administered with multimodal analgesia. There was also a significant main effect of time (X^2^ = 207.21, *p* < 0.001) where rabbits exhibited significantly more pain behaviors at 1 h post-surgery compared to all other morning time points. Similarly, for the afternoon observations, a main effect of treatment (X^2^ = 5.0337, *p* = 0.025) and time (X^2^ = 63.369, *p* < 0.001) were found, with significantly more pain behaviors exhibited by meloxicam only treated rabbits in comparison to multimodal and significantly more pain behaviors displayed at 5 h post-surgery than the baseline afternoon time point (Figure 2B).

#### Batch 2 (New Zealand White)

**Figure 3:**
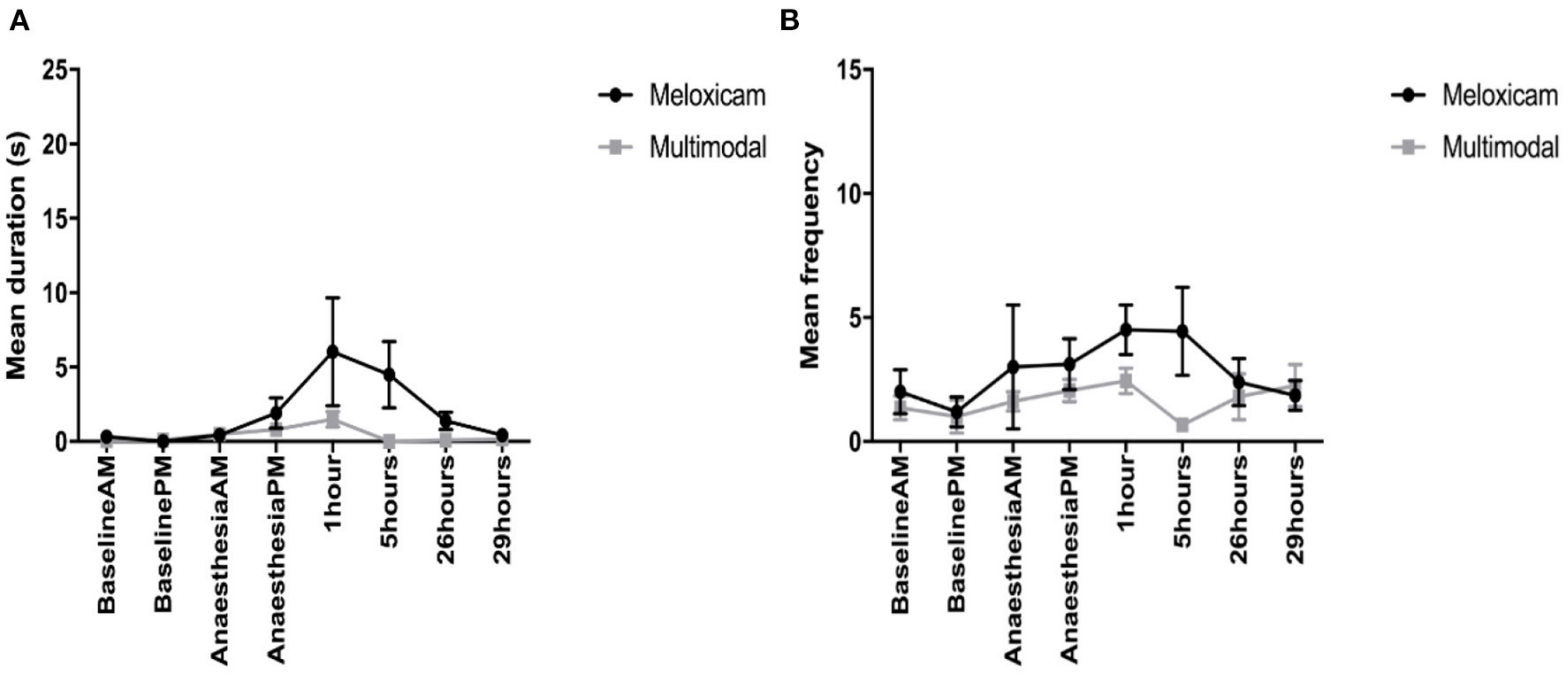
**(A)** The mean (±SEM) duration (s) of pain behaviors (arching, pressing, falling, writhing, and shuffling) over time, in New Zealand White rabbits administered meloxicam alone or a multimodal analgesic regime (*n* = 8 per treatment group). **(B)** The mean (±SEM) frequency of pain behaviors (arching, falling, flinching, twitching, pressing, staggering, wincing, writhing, and shuffling) over time, in New Zealand White rabbits administered meloxicam alone or a multimodal analgesic regime (*n* = 8 per treatment group).

For the duration of time spent displaying active pain behaviors (composite pain duration) across the morning observations (Baseline AM, AA-AM, and 1 and 24 h post-surgery), those rabbits administered meloxicam alone displayed significantly more active pain behaviors than those in the multimodal treatment group at 1 h post-surgery (X^2^ = 12.768, *p* = 0.03). There was also an effect of time, irrespective of treatment group (X^2^ = 34.746, *p* < 0.001), with rabbits displaying more active pain behaviors at 1 h post-surgery compared to Baseline AM, AA-AM, and 24 h post-surgery. For the duration of active pain behaviors during the afternoon observations (Baseline PM, AA-PM, 5 and 29 h post-surgery), there was a significant interaction between time and treatment (X^2^ =19.323, *p* = <0.001) and a main effect of time (X^2^ = 14.149, *p* = <0.001). *Post-hoc* analysis showed that rabbits administered meloxicam alone displayed active pain behaviors for significantly longer than those in the multimodal treatment group at 5 h post-surgery and spent significantly longer displaying these behaviors compared to Baseline PM (Figure 3A).

For the frequency of pain behaviors observed (composite pain frequency) during the morning observations, the only factor found to have a main effect was time (X^2^ = 8.159, *p* = 0.043). Although Tukey *post-hoc* analysis showed no significant differences between any individual pairwise comparisons, there was a trend toward rabbits displaying more pain behaviors at 1 h post-surgery compared to baseline AM observations. For the afternoon time observations, there was a significant interaction of time and treatment interaction (X^2^ = 10.794, *p* = 0.013). Tukey *post-hoc* analysis showed that rabbits administered meloxicam exhibited more pain behaviors at 5 h post-surgery compared to rabbits administered multimodal analgesia (Figure 3B).

### Rabbit Grimace Scale

#### Still Image Scoring—Batch 1 (Dutch Belted)

**Figure 4:**
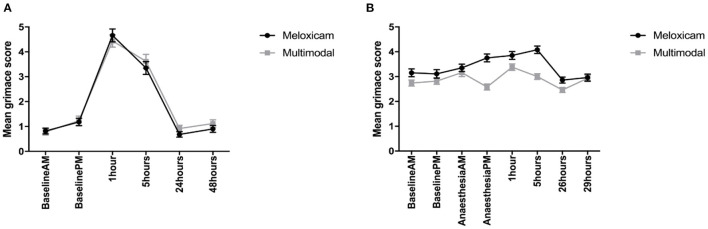
**(A)** Mean (±SEM) rabbit grimace scale scores over time, in Dutch Belted rabbits, obtained from still images (*n* = 8 per treatment group). **(B)** Mean (±SEM) rabbit grimace scale scores over time, in New Zealand White rabbits, obtained from still images (*n* = 8 per treatment group).

For the mean total RbtGS scores during the morning observation time points (Baseline AM, and 1, 24, and 48 h post-surgery), there was no effect of treatment, but there was a significant effect of time (X^2^ = 608.42, *p* = <0.001), and a time treatment interaction (X^2^ = 17.461, *p* < 0.001). Tukey *post-hoc* tests revealed that irrespective of treatment group, the RbtGS scores were greater at 1 h post-surgery than at any of the other morning time points. Rabbits administered meloxicam alone had a greater RbtGS score at 1 h post-surgery than any other morning time point. For the afternoon observations (Baseline PM and 5 h post-surgery), the total mean RbtGS scores were not significantly different between the two analgesic treatment groups. However, there was a significant effect of time (X^2^ = 131.68, *p* < 0.001). Rabbits had a significantly higher RbtGS score at 5 h post-surgery than at Baseline PM (Figure 4A).

#### Still Image Scoring—Batch 2 (New Zealand White)

For the mean total RbtGS scores during the morning observations (Baseline AM, AA-AM, 1 and 24 h post-surgery), there was no effect of treatment, but there was a significant effect of time (X^2^ = 72.348, *p* = <0.001) and a significant time treatment interaction (X^2^ = 96.751, *p* < 0.001). Irrespective of treatment, rabbits had the greatest RbtGS score at 1 h post-surgery compared to AA-AM. However, RbtGS scores were greater at both these time points compared to Baseline AM and 24 h post-surgery. Rabbits that had received meloxicam alone had significantly greater RbtGS scores one post-surgery compared to all other morning time points. In contrast, rabbits administered multimodal analgesia had a significantly lower RbtGS score at 1 h post-surgery compared to Baseline AM (Figure 4B).

For the mean total RbtGS scores during the afternoon observations (Baseline PM, AA-PM, and 5 and 29 h post-surgery), there was a significant main effect of both treatment (X^2^ = 4.7962, *p* = 0.029) and time (X^2^ = 82.281, *p* < 0.001). Tukey *post-hoc* analysis revealed that irrespective of treatment group, rabbits had higher RbtGS scores at AA-PM and 5 h post-surgery compared to Baseline PM and 29 h post-surgery. Rabbits administered meloxicam alone had greater grimace scale scores at surgery +5 h compared to Baseline PM and surgery +29 h. Those rabbits administered multimodal analgesia did not have greater grimace scale scores following surgery (Figure 4B).

#### Video Scoring—Batch 1 (Dutch Belted)

**Figure 5:**
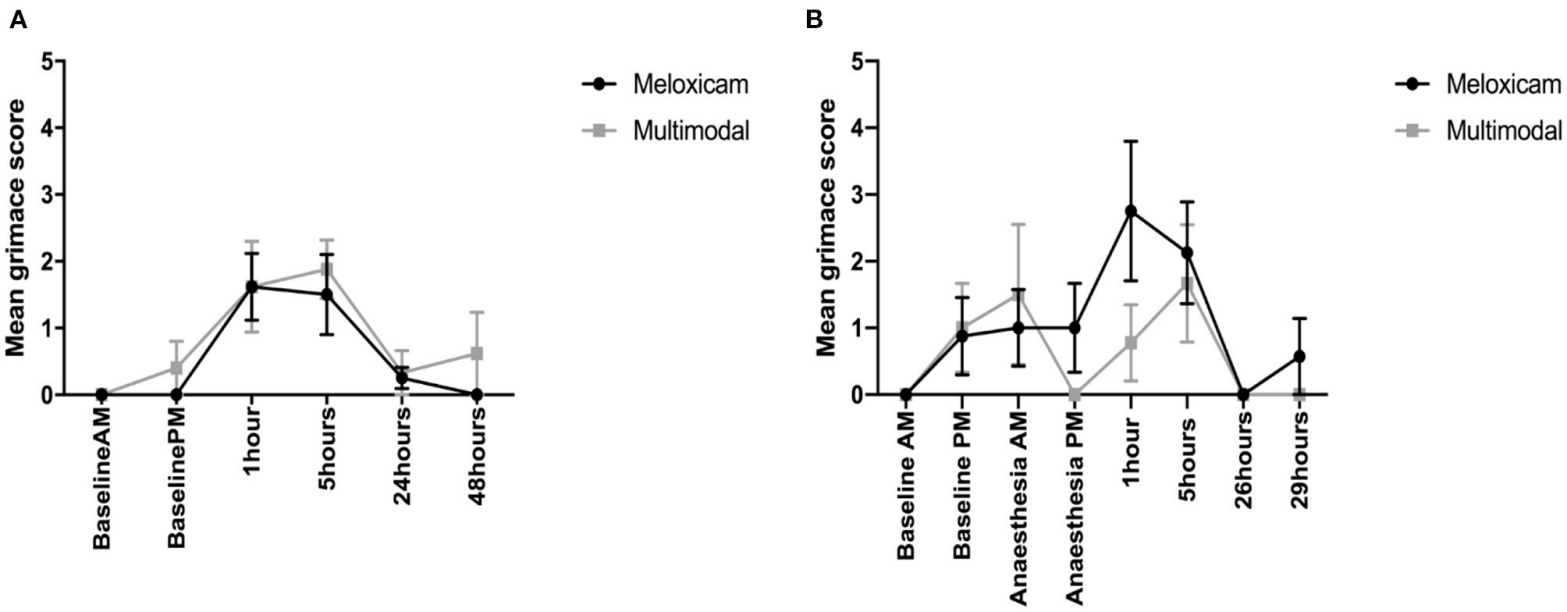
**(A)** Mean (±SEM) rabbit grimace scale scores over time, in Dutch Belted rabbits, obtained from video sequences (*n* = 8 per treatment group). **(B)** Mean (±SEM) rabbit grimace scale scores over time, in New Zealand White rabbits, obtained from video sequences (*n* = 8 per treatment group).

When comparing RbtGS scores, from video recordings, across the morning time points, the only factor shown to have a significant effect was time (X^2^ = 30.812, *p* < 0.001), with no significant difference in the RbtGS scores between the treatment groups. Tukey *post-hoc* analysis showed that the rabbits were assigned higher grimace scale scores at surgery +1 h compared to all other morning time points. When analysing the afternoon time point data, a similar pattern was seen, where the only factor to show a significant effect was time (X^2^ = 13.146, *p* < 0.001), with a higher grimace score at surgery + 5 h compared to Baseline PM (see Figure 5A).

#### Video Scoring—Batch 2 (New Zealand White)

For the mean total RbtGS scores during the morning observations, there was a significant effect of time (X^2^ = 26.079, *p* < 0.001) and a significant time treatment interaction (X^2^ = 8.0708, *p* = 0.045). Tukey *post-hoc* analysis showed that rabbits administered meloxicam alone had a significantly higher RbtGS score at 1 h post-surgery compared to Baseline AM and 24 h post-surgery. There were no significant differences in the rabbits administered multimodal analgesia. For the afternoon time observations, time was the only factor to have a significant effect (X^2^ = 13.865, *p* = 0.003), with rabbits assigned higher RbtGS scores at 5 h post-surgery compared to all other PM time points (see Figure 5B).

### Bodyweight

#### Batch 1 (Dutch Belted)

No significant difference was found in the bodyweight of the rabbits over time or between the two analgesic treatment groups at any time point.

#### Batch 2 (New Zealand White)

No significant difference was found between the bodyweight of the rabbits in the two treatment groups at any time point. A significant difference in body weight was observed over time (X^2^ = 27.23, *p* < 0.001), with rabbits found to be significantly heavier 48 h post-surgery, compared to both baseline and 24 h post-surgery.

### Food and Water Consumption

#### Batch 2 (New Zealand White)

**Figure 6:**
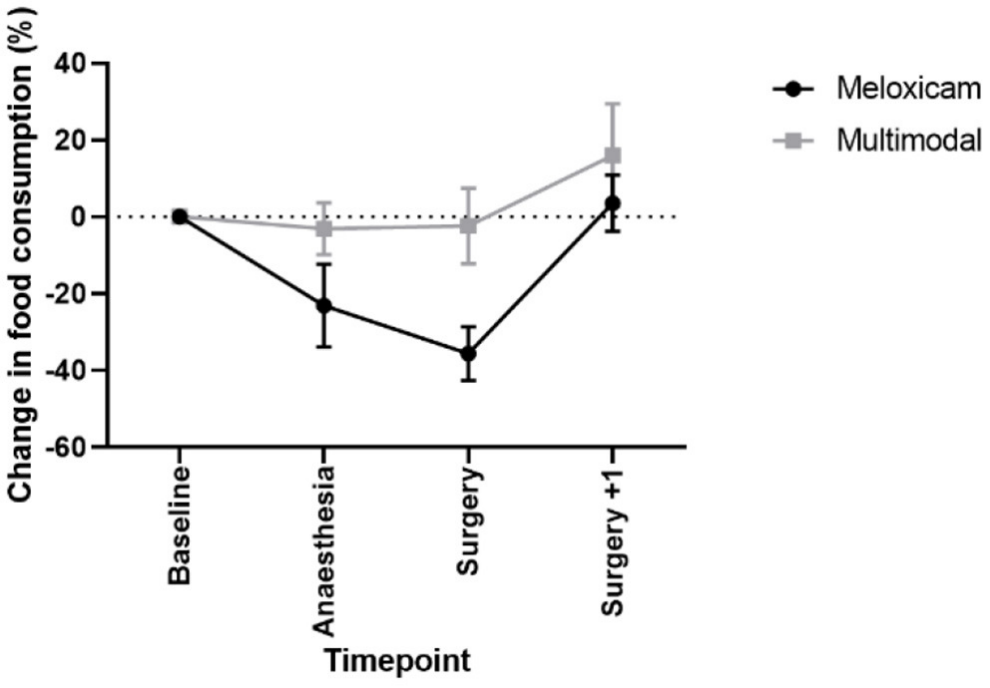
Mean % change in food consumption (±SEM) in New Zealand White rabbits at baseline, following anesthesia and analgesia alone, the day of castration and one-day post castration.

There was a significant effect of treatment (X^2^ = 3.807, *p* = 0.05), with rabbits receiving meloxicam alone showing a greater reduction in food consumption relative to baseline levels than those rabbits administered multimodal analgesia. A significant effect of time was also found (X^2^ = 17.756, *p* < 0.001; see Figure 6), with lower food consumption compared to baseline immediately following surgery which recovered post-surgery (surgery+1). This was irrespective of analgesic treatment, we found a non-significant time x treatment interaction (X^2^ = 5.78, *p* = 0.123). No significant difference was observed between any time points or treatment groups in the volume of water consumed.

### Dark Phase Activity

#### Batch 2 (New Zealand White)

No significant difference was found between any time points or treatment groups in the number of zone transitions made during the dark phase.

## Discussion

Rabbits are the third most common pet in UK households following dogs and cats (1) and around 11,000 are used per annum in scientific research (2). Many of the rabbits kept as companion animals will undergo at least one potentially painful procedure during their lifetime, with castration being one of the most common procedures. Rabbits used in research procedures may also undergo surgical procedures as necessary parts of research protocols. To be able to reduce or alleviate pain following surgery effectively, we must be able to accurately assess its presence and be able to do so rapidly at the pen or cage side.

Manual scoring of behavior is commonly used in the assessment of pain and analgesic efficacy in animals [e.g., (14, 29–31)]. Following the identification of key species and procedure-specific pain behaviors, animals can be monitored over extended periods to determine the efficacy of analgesic regimens and to identify times when further treatment is required. This approach is time-consuming but is often used for research purposes, using video recordings. In a clinical setting, live scoring of behavior in rabbits is particularly difficult due to their tendency to freeze in the presence of an observer. Our first objective was to determine if the rabbit grimace scale (RbtGS) offered an effective alternative to manual scoring of behavior, both from still images and from video footage. If scoring from video footage proved to be successful, a live feed could be recommended to allow remote monitoring of rabbits to allow evaluation without inducing freezing behavior. Following surgery, both Dutch Belted and New Zealand White rabbits showed significant increases in the duration spent displaying key pain associated behaviors compared to baseline levels. These changes were in line with those reported by Leach et al. (14) following ovariohysterectomy and were present at 1 and 5 h post-surgery. RbtGS scoring carried out at the same time using still images (remotely recorded), showed the same pattern of changes in both breeds of rabbit, with significantly higher RbtGS scores recorded at 1 and 5 h post-surgery compared to baseline. Scoring using the RbtGS is significantly faster, and staff can be trained to accurately score grimace scales rapidly (19, 32) making this a potentially useful addition to behavioral analysis. This could allow key time periods when rabbits require analgesic intervention to be identified more quickly. The RbtGS was also scored from video footage (rather than still images) to simulate pain assessment using a remote viewing technique. In line with the behavioral analysis, Dutch belted rabbits showed an increase in grimace scale score at both 1 and 5 h post-surgery. However, when scoring the New Zealand White rabbits, an increase in score was only observed at 1 h post-surgery, indicating that in this breed the RbtGS was not capable to replicating the pain score demonstrated by full behavioral scoring beyond the 1 h post-surgery. Response to nociceptive stimuli and analgesia has been widely studied and variation between strains within a species is a significant finding (33–35). Moreover, Miller and Leach (32) found significant differences in baseline grimace scale scores between strains of mice, indicating the importance of determining baseline scores for specific groups of animals, before using this methodology for pain assessment. Given the propensity of rabbits to freeze in the presence of humans (14), remote scoring must be investigated both in terms of directly viewing a video for assessment and retrospective scoring of images for evaluation of new analgesic regimes when using multiple observers blinded to treatment. While this is not always feasible in a clinical setting, for routine procedures such as orchidectomy, baseline measures can be taken to allow the most valid comparison to post-surgery grimace scores. Use of the RbtGS coupled with observation via a web-cam or video recording could enable rapid pain assessment in the immediate hours following surgery and adjustment of the analgesic regimen to provide effective pain control.

Additionally, we compared the use of multimodal analgesic regimens to an earlier recommended dose of meloxicam alone (0.2 mg/kg). The dose of meloxicam (0.6 mg/kg) used in the multi modal approach was higher than that of the meloxicam alone group, as we considered that improved analgesia could be achieved by doing so. We aimed to compare the use of the RbtGS with a range of analgesic regimens, thought likely to produce varying degrees of post-operative analgesia. Batch 1 evaluated the combination of meloxicam with a lidocaine/bupivacaine infiltration of the surgical site in Dutch belted rabbits. Batch 2 studied the combination of meloxicam and buprenorphine with a lidocaine/bupivacaine infiltration of the surgical site in New Zealand White rabbits. In Batch 1, Dutch belted rabbits that had received a lower dose of meloxicam alone showed significantly more pain behaviors at 1 h post-surgery and 5 h post-surgery compared to those that had been administered a combination of a higher dose of meloxicam and a lidocaine/bupivacaine local infusion. When the RbtGS was scored using still images, a treatment difference was only observed at 1 h post-surgery, suggesting that in its current format, the RbtGS may only be of benefit for comparing analgesics in the most acute phase of post-operative pain for this breed. However, no difference was seen between the two treatment groups at any time point when scoring the RbtGS from a video (simulating a clinical setting), despite differences being observed in the behavior of the rabbits. In Batch 2, the New Zealand white rabbits that had received the lower dose of meloxicam alone displayed pain behaviors for a significantly longer period than those that had received a combination of higher dose meloxicam and buprenorphine with a lidocaine/bupivacaine local infiltration at 1 and 5 h post-surgery. When assessed using the rabbit grimace scale, scores from still images and video were higher in the lower dose meloxicam only group than the multimodal group at 1 and 5 h post-surgery. This finding replicates the pattern of results obtained from the behavioral analysis, indicating that in this particular breed of rabbits, the RbtGS was effective at determining time points when pain is likely being experienced both in research and clinical settings. The multimodal analgesic regimen administered differed between the two breeds of rabbits. In Batch 1, meloxicam was administered alongside a lidocaine/bupivacaine local infiltration. Following this batch, we subjectively felt that the multi modal approach could be further refined, so in Batch 2, the same approach was taken but with the addition of 0.03 mg/kg buprenorphine. While breed differences cannot be ruled out because of the study design, those rabbits that received buprenorphine did not show an decrease in RbtGS score using either method of assessment.

Across the time points studied, changes in behavior were only observed in the first 5 h following surgery. The next time point studied for full behavioral analysis was 24 h post-surgery when no significant differences were observed in pain behaviors compared to baseline. During this gap in behavioral analysis, dark phase activity was monitored in the New Zealand white rabbits. The methodology used was to count the number of transitions between zones in the pen, and no significant differences were found either between groups or between baseline and post-surgery time points. Further research is required to determine whether dark phase activity measured in this way was not sensitive enough to detect behavioral changes linked to pain following surgery or whether it was used beyond the period in which rabbits showed acute changes related to pain. Future research should focus on the time frame between 5 and 24 h post-surgery to determine the overall length of time pain is likely to be experienced by rabbits following orchidectomy.

## Conclusion

Using behavioral analysis as the “gold standard” for comparison, the RbtGS was an effective means of determining when rabbits are experiencing acute pain following orchidectomy. RbtRGS differentiated the effects of low dose meloxicam (0.2 mg/kg) from that achieved with a higher dose of meloxicam (0.6 mg/kg) combined with either buprenorphine (0.03 mg/kg) and local anesthetic infiltration (in New Zealand White rabbits) or local anesthetic infiltration (in Dutch Belted rabbits). The combinations using a higher dose of meloxicam were more effective in reducing pain compared to the lower dose of meloxicam alone.

## Data Availability Statement

The raw data supporting the conclusions of this article will be made available by the authors, without undue reservation.

## Ethics Statement

All procedures were conducted in accordance with the Animals (Scientific Procedures) Act 1986, European Directive 2010/63 and with the approval of the Newcastle University Animal Welfare Ethical Review Body.

## Author Contributions

AM, ML, PF, and JC: conception, design of the work, and interpretation of data. AM, ML, PF, JC, CQ, VN, CK, and AG-S: acquisition and analysis. All authors contributed to the article and approved the submitted version.

## Funding

This project was funded by NC3Rs, grant number: G1100563/1 Newcastle University provided funds for open access publication of this manuscript.

## Conflict of Interest

The authors declare that the research was conducted in the absence of any commercial or financial relationships that could be construed as a potential conflict of interest.

## Publisher's Note

All claims expressed in this article are solely those of the authors and do not necessarily represent those of their affiliated organizations, or those of the publisher, the editors and the reviewers. Any product that may be evaluated in this article, or claim that may be made by its manufacturer, is not guaranteed or endorsed by the publisher.
